# A Longitudinal Study of Structural Risk Factors for Obesity and Diabetes Among American Indian Young Adults, 1994–2008

**DOI:** 10.5888/pcd12.140469

**Published:** 2015-05-07

**Authors:** Tennille L. Marley, Molly W. Metzger

**Affiliations:** Author Affiliation: Tennille L. Marley, Arizona State University, Tempe, Arizona.

## Abstract

**Introduction:**

American Indian young adults have higher rates of obesity and type 2 diabetes than the general US population. They are also more likely than the general population to have higher rates of structural risk factors for obesity and diabetes, such as poverty, frequent changes of residence, and stress. The objective of this study was to investigate possible links between these 2 sets of problems.

**Methods:**

Data from the American Indian subsample of the National Longitudinal Study of Adolescent to Adult Health (Add Health) were used to examine potential links between obesity and type 2 diabetes and structural risk factors such as neighborhood poverty, housing mobility, and stress. We used logistic regression to explore explanatory factors.

**Results:**

American Indians in the subsample had higher rates of poor health, such as elevated hemoglobin A1c levels, self-reported high blood glucose, self-reported diabetes, and overweight or obesity. They also had higher rates of structural risk factors than non-Hispanic whites, such as residing in poorer and more transient neighborhoods and having greater levels of stress. Self-reported stress partially mediated the increased likelihood of high blood glucose or diabetes among American Indians, whereas neighborhood poverty partially mediated their increased likelihood of obesity.

**Conclusion:**

Neighborhood poverty and stress may partially explain the higher rates of overweight, obesity, and type 2 diabetes among American Indian young adults than among non-Hispanic white young adults. Future research should explore additional neighborhood factors such as access to grocery stores selling healthy foods, proximity and safety of playgrounds or other recreational space, and adequate housing.

## Introduction

Rates of overweight, obesity, and type 2 diabetes are growing in the United States across all racial and ethnic groups and among children and adolescents (1–4). However, American Indian adolescents and young adults are more likely than adolescents and young adults of other races and ethnicities to have these conditions (4,5). American Indian adolescents are more likely to be overweight or obese (42%) than non-Hispanic whites (26.7%), Latinos (37.6%), and African Americans (41.1%) (2). From 1990 to 1998, type 2 diabetes diagnoses increased by 71% among American Indian children, adolescents, and young adults and prevalence increased by 68% (from 1.23 per 1,000 to 5.42 per 1,000) among American Indian adolescents aged 15 to 19 (5). Overweight and obesity can have serious consequences for health, including cardiovascular disease, type 2 diabetes, and other conditions that can contribute to lower quality of life, disability, and premature death (6).

Structural determinants and conditions of daily life make up the social determinants of health and are responsible for many poor health outcomes, and increasingly, researchers recognize the effects of various social determinants that contribute to overall health (7–9). A growing body of research suggests that disease and ill health are largely the result of the “circumstances in which people are born, grow, live, work, and age, and the systems put in place to deal with illness” (9). Despite evidence of the associations among social determinants and health, empirical research on possible links between neighborhood factors and obesity, overweight, and type 2 diabetes among American Indians is scarce (3,10–13). The objective of this study was to explore the associations between the structural determinants of neighborhood factors, parent education and obesity, and perceived stress with overweight, obesity, and type 2 diabetes among American Indian young adults.

## Methods

### Survey design

This study used data from the first and fourth waves of the National Longitudinal Study of Adolescent to Adult Health (Add Health), a nationally representative study following adolescents into early adulthood (14). These waves were chosen to capitalize on the rich neighborhood data available in Wave 1 and the multiple outcomes related to type 2 diabetes and obesity in Wave 4.

Wave 1 comprised adolescents in grades 7 through 12 (ages 12–19) in school year 1994–1995. Participants originated from a stratified random sample of 20,745 adolescents attending 80 high schools and 52 middle schools. The schools were stratified into 80 clusters, by variables such as region (Northeast, Midwest, South, West), urbanicity (urban, suburban, rural), school type (public, private, parochial), and other characteristics. In addition to the surveys of the adolescents themselves, 17,670 parents also completed interviews at Wave 1. Wave 4 followed up with those adolescents when they were young adults aged 24 to 32 in 2007–2008. The Wave 4 follow-up included 76% of the original sample (n = 15,701).

Attrition between Waves 1 and 4 differed by race and ethnicity. The Wave 4 response rate was highest for white participants (79%) and lowest for Asian participants (66%). The response rate was slightly below average for American Indian participants (73%). To adjust for this differential response, all analyses used Add Health’s longitudinal sampling weights designed for the Wave 4 sample (“pweights” in Stata [StataCorp LP]). These weights adjust for complex sample design, selection, and nonresponse, including adjustment for differential response by race, education level, and marital status (15). Overall, complete data were available for 12,657 respondents. 

### Measures

#### Dependent variables

Analyses included 4 outcomes: glycated hemoglobin (HbA1c), self-reported high blood glucose or type 2 diabetes, overweight/obesity, and obesity. For HbA1c, whole-blood spot assays were collected via finger pricks, and levels were determined from colorimetric methods. HbA1c values greater than 5.7 were considered elevated.

In addition to the direct measure of HbA1c, we examined self-reports of high blood glucose or type 2 diabetes, measured with a single item, “Has a doctor, nurse, or other health care provider ever told you that you have or had high blood sugar or diabetes?” We used direct measures of height and weight to calculate body mass index (BMI, kg/m^2^). Overweight/obesity was defined as a BMI greater than or equal to 25.0 and obesity as a BMI greater than or equal to 30.0.

#### Race and ethnicity

Racial and ethnic classifications were based on self-report at Wave 1. Our primary group of interest, American Indians, included participants who selected “Native American” solely or in combination with another racial or ethnic group. The Hispanic category included those self-reporting as Hispanic solely or in combination with another group (not including Native American). The white, black, and Asian categories comprised those self-reporting as each of those groups not in combination with another group. Because of small sample sizes, participants self-reporting as other combinations of racial and ethnic groups were classified as “other.”

#### Context measures

Three measures of neighborhood characteristics were included in the analyses: neighborhood collective efficacy (ie, social cohesion), neighborhood poverty rate, and neighborhood mobility. Neighborhood collective efficacy was reported by adolescents in Wave 1 (16) and was calculated as the sum of 3 dichotomous (true/false) items: “You know most of the people in your neighborhood,” “In the past month, you have stopped on the street to talk with someone who lives in your neighborhood,” and “People in this neighborhood look out for each other.” For each item, a no response was scored as zero, and a yes response was scored as 1. Data for the second and third neighborhood measures were from 1990 Census block group data. Neighborhood poverty rate is the percentage of people living below the official poverty threshold ($13,254 for a family of 4 in 1990). “Neighborhood mobility” was measured as the percentage of occupied housing units into which people moved during the previous 5 years. In addition to the neighborhood measures, dummy variables were included to reflect participants’ school location in suburban, rural, or urban locations at Wave 1.

#### Additional variables

A robust set of control variables and mediators was included. Control variables from Wave 1 were adolescent-reported age, sex, and parent’s highest level of educational attainment (for 2-parent families, data were used for the parent with the higher level of education). From Wave 4, we included the Cohen Perceived Stress Scale (PSS) (17,18). The PSS score was calculated as the sum of 4 items (range, 0–16). Participants reported how often during the previous 30 days they 1) were unable to control important things in their lives, 2) felt confident in their ability to handle their personal problems (reversed), 3) felt things were going their way (reversed), and 4) felt that difficulties were piling up so high that they were unable to overcome them. Each item was scored as 0 (never), 1 (almost never), 2 (sometimes), 3 (fairly often), or 4 (very often).

### Statistical analysis

We calculated descriptive statistics (percentages, means, and 95% confidence intervals) for the full sample and American Indian subsample. We calculated adjusted odds ratios (AORs) and *P* values (significance set at an α level of .05) from a series of logistic regression models predicting elevated HbA1c, self-reported high blood glucose, and self-reported diabetes. Logistic regression models, neighborhood predictors, and perceived stress were entered as *z* scores for ease of comparison across coefficients. Finally, Sobel tests were conducted as a test of mediation. All analyses were implemented in Stata version 12 (StataCorp LP). Procedures for data access and analysis were implemented as approved by the institutional review board at Northwestern University and in agreement with the sensitive data security plan approved by Add Health data managers.

## Results

Our analytic sample comprised 11,110 participants, including 393 participants who self-identified as American Indian (Table 1). At Wave 1, the full sample resided in neighborhoods with a poverty rate of 13.9%, whereas the American Indian subsample resided in neighborhoods with an average neighborhood poverty rate of 19.2%. Neighborhood mobility was higher for the American Indian subsample than for the full sample; 49.3% in the subsample and 46.5% in the full sample of neighbors resided in the neighborhood for less than 5 years. The mean score for neighborhood collective efficacy was 0.75 for both the American Indian subsample and the full sample. At Wave 4, the mean score on the Cohen PSS was higher among American Indians (score, 5.6) than among the full sample (score, 4.8.)

**Table 1:** Descriptive Statistics for Full Sample and American Indian Subsample, National Longitudinal Study of Adolescent to Adult Health, 1994–2008

Characteristic	Full Sample (n = 11,110)	American Indian Subsample (n = 393)
**Neighborhood (Wave 1), mean (95% CI)**		
Neighborhood collective efficacy^a^	0.75 (0.74–0.77)	0.75 (0.71–0.79)
Neighborhood poverty^b^	13.9 (12.2–15.6)	19.2 (13.2–25.1)
Neighborhood mobility^c^	46.5 (45.0–48.0)	49.3 (46.5–52.0)
**Urbanicity, % (95% CI)**		
Urban	25.4 (18.5–33.9)	32.8 (21.0–47.3)
Suburban	58.2 (48.2–67.5)	45.7 (31.1–61.1)
Rural	16.4 (9.5–26.9)	21.4 (8.3–45.0)
**Family (Wave 1), % (95% CI)**		
Parent has ≥high school diploma	86.3 (83.9–88.4)	80.2 (73.6–85.5)
Parent has ≥college diploma	33.3 (30.0–36.9)	18.2 (13.2–24.6)
Parent is obese	22.9 (21.7–24.1)	36.3 (29.8–43.3)
**Individual stress and health (Wave 4)**		
Perceived stress, mean score^d^ (95% CI)	4.8 (4.7–4.9)	5.6 (5.2–6.0)
HbA1c value, mean (95% CI)	5.6 (5.5–5.6)	5.7 (5.6–5.8)
HbA1c ≥5.7, % (95% CI)	30.6 (28.4–32.8)	43.8 (36.4–51.5)
Ever told have high blood glucose or diabetes, % (95% CI)	2.6 (2.2–3.0)	5.2 (2.8–9.3)
Mean body mass index, kg/m^2^ (95% CI)	29.1 (28.8–29.5)	30.7 (29.0–32.3)
Overweight or obese, % (95% CI)	66.6 (64.9–68.3)	76.8 (69.9–82.6)
Obese, % (95% CI)	37.4 (35.5–39.2)	42.5 (33.7–51.8)

Abbreviations: HbA1c, glycated hemoglobin; CI, confidence interval.

a A measure of social cohesion scored on a scale of 0 to 3, with a higher score indicating better neighborhood efficacy.

b Percentage of people living below the official poverty threshold, based on 1990 Census block group data.

c Measured as the percentage of occupied housing units into which people moved during the previous 5 years, based on1990 Census block group data.

d Cohen Perceived Stress Scale (PSS) (17,18). Scored on a scale of 0 to 16, with higher scores indicating greater stress.

The American Indian subsample was more likely than the full sample to have health problems across multiple indicators at Wave 4: 43.8% of the American Indian subsample had elevated HbA1c levels, compared with 30.6% of the full sample; 5.2% of the American Indian subsample reported having been told they had high blood glucose or type 2 diabetes, compared with 2.6% of the full sample; 76.8% of the American Indian subsample was overweight/obese or obese, compared with 66.6% of the full sample; and 42.5% of the American Indian sample was obese, compared with 37.4% of the full sample.

All racial/ethnic minority groups included in our logistic regression models were more likely than non-Hispanic whites to have elevated HbA1c (Table 2). In Model 1 (no control variables), American Indians were 2.66 times as likely as non-Hispanic whites to have elevated HbA1c (*P* < .01); in Model 2 (controls for sex, age, parent education, and parent obesity), they were 2.47 times as likely (*P* < .01). In Model 3 (further addition of controls for neighborhood variables, urbanicity, and perceived stress), the adjusted odds ratio (AOR) of American Indians having elevated HbA1c was further attenuated to 2.41; one of the 3 neighborhood variables (neighborhood collective efficacy) was significantly associated with elevated HbA1c (AOR, 1.07; *P* = .04); perceived stress was not. In Model 4 (addition of overweight/obesity and obesity), both overweight/obesity and obesity predicted elevated HbA1c (*P* < .01 for both). In this model, the likelihood of elevated HbA1c among American Indians was attenuated with the inclusion of overweight/obesity and obesity but remained significant (AOR, 2.38; *P* < .01). Post hoc tests showed that overweight/obesity and obesity may partially mediate the relationship between being American Indian and having elevated HbA1c (Sobel *z* = 2.42, *P* = .02 for overweight/obesity; Sobel* z* = 1.83, *P* = .07 for obesity).

**Table 2:** Adjusted Odds Ratios From Logistic Regression Models of HbA1c and Self-Reported High Blood Glucose or Diabetes Among Young Adults (n = 11,110), National Longitudinal Study of Adolescent to Adult Health, 1994–2008

Characteristic	HbA1c (Direct Measurement)					Diagnosis of High Blood Glucose or Diabetes (Self-Reported)			
	Model 1		Model 2	Model 3	Model 4	Model 5	Model 6	Model 7	Model 8
**Race/ethnicity**									
White [Reference]	1.00		1.00	1.00	1.00	1.00	1.00	1.00	1.00
American Indian	2.66^a^		2.47^a^	2.41^a^	2.38^a^	2.39^b^	1.95	1.83	1.82
Black	4.86^a^		4.94^a^	4.68^a^	4.62^a^	1.48^b^>	1.31	1.19	1.10
Hispanic	2.20^a^		2.07^a^	2.15^a^	2.00^a^	1.57	1.25	1.26	1.13
Asian	2.06^a^		2.30^a^	2.42^a^	2.69^a^	0.43	0.50	0.50	0.58
Other race/ethnicity	2.25^a^		2.33^a^	2.38^a^	2.44^a^	0.71	0.66	0.65	0.62
**Male**	—		1.78^a^	1.78^a^	1.80^a^	—	0.74	0.78	0.80
**Age**	—		1.07^a^	1.08^a^	1.07^a^	—	1.08	1.08	1.07
**Parent education**									
<High school diploma [Reference]		—	1.00	1.00	1.00	—	1.00	1.00	1.00
High school diploma		—	0.82^b^	0.83^b^	0.82^b^	—	0.60	0.64	0.63
Some college		—	0.72^a^	0.75^a^	0.76^a^	—	0.56^b^	0.61	0.63
College diploma		—	0.63^a^	0.66^b^	0.70^a^	—	0.40^a^	0.45^a^	0.49^a^
>College diploma		—	0.61^a^	0.65^a^	0.71^b^	—	0.28^a^	0.32^b^	0.38
**Parent is obese**		—	1.52^a^	1.52^a^	1.25^a^	—	1.72^a^	1.70^a^	1.36
**Neighborhood characteristics^c^**									
Neighborhood collective efficacy^d^		—	—	1.07^b^	1.07^b^	—	—	1.02	1.01
Neighborhood poverty^e^		—	—	1.06	1.03	—	—	1.04	1.00
Neighborhood mobility^f^		—	—	1.00	1.01	—	—	0.92	0.93
**Urbanicity**									
Suburban [Reference]		—	—	1.00	1.00	—	—	1.00	1.00
Rural		—	—	1.14	1.14	—	—	0.95	0.94
Urban		—	—	.96	1.00	—	—	1.09	1.14
Perceived stress^c^^, ^ g		—	—	1.01	1.01	—	—	1.09^a^	1.09^a^
**Weight status**									
Neither overweight or obese [Reference]		—	—	—	1.00	—	—	—	1.00
Overweight/obesity		—	—	—	1.47^a^	—	—	—	1.03
Obese		—	—	—	2.88^a^	—	—	—	3.47^a^
**Constant**		0.29	0.09	0.07	0.05	0.03	0.01	0.01	0.004

Abbreviation: —, not applicable; HbA1c, glycated hemoglobin.

a *P* < .01.

b *P* < .05.

c Calculated as *z* scores, normed such that the mean equals zero and standard deviation equals 1. Coefficients can be interpreted as the adjusted odds ratio associated with a 1 standard deviation increase in the predictor.

d A measure of social cohesion scored on a scale of 0 to 3, with a higher score indicating better neighborhood efficacy.

e Percentage of people living below the official poverty threshold, based on 1990 Census block group data.

f Measured as the percentage of occupied housing units into which people moved during the previous 5 years, based on1990 Census block group data.

g Measure of stress based on the Cohen Perceived Stress Scale (PSS) (17,18). Scored on a scale of 0 to 16, with higher scores indicating greater stress.

In Model 5 (Table 2), American Indians were 2.39 times as likely as non-Hispanic whites to self-report high blood glucose or diabetes (*P* = .02), similar to the findings for elevated HbA1c. However, compared with the control variables for HbA1c, the control variables for high blood glucose and diabetes mediated associations more strongly. In Model 6 (controls for sex, age, parent education, and parent obesity), the AOR for American Indians decreased 1.95 (*P* = .07); in Model 7 (further addition of controls for neighborhood variables, urbanicity, and perceived stress), the AOR decreased to 1.83 (*P* = .12), and in Model 8 (addition of overweight/obesity and obesity), it further decreased to 1.82 (*P* = .14). Although none of the 3 neighborhood indicators was significantly associated with high blood glucose or diabetes, perceived stress was (AOR, 1.09; *P* < .01). One standard deviation increase in perceived stress was associated with a 9% increase in the likelihood of high blood glucose or diabetes, and post hoc tests confirmed perceived stress as a mediator (Sobel *z* = 2.23; *P* = .03). Obesity did not mediate the association between being American Indian and self-reporting high blood glucose or diabetes (Sobel *z *= 1.76, *P* = .08).

In models predicting overweight/obesity or obesity (Models 1–3) and obesity (Models 4–6) (Table 3), American Indians were more likely than non-Hispanic whites to be overweight/obese or obese, and this association was attenuated by the inclusion of covariates. In Model 6 (controls for all variables), neighborhood poverty was significantly associated with obesity (AOR, 1.17, *P* < .01). Neighborhood poverty was a partial mediator of the association between being American Indian and being obese (Sobel *z*, 2.01; *P* = .05)

**Table 3:** Adjusted Odds Ratios From Logistic Regression Models of Overweight and Obesity, National Longitudinal Study of Adolescent to Adult Health (n = 11,110), 1994–2008

Characteristic	Overweight/Obesity			Obesity		
	Model 1	Model 2	Model 3	Model 4	Model 5	Model 6
**Race/ethnicity**						
White [Reference]	1.00	1.00	1.00	1.00	1.00	1.00
American Indian	1.87^a^	1.62^b^	1.65^a^	1.38^b^	1.15	1.09
Black	1.54^a^	1.51^a^	1.45^a^	1.63^a^	1.58^a^	1.34^a^
Hispanic	1.75^a^	1.70^a^	1.78^a^	1.48^a^	1.43^a^	1.47^a^
Asian	0.67	0.81	0.84	0.51^a^	0.64^b^	0.67
Other race/ethnicity	0.96	0.94	0.96	1.11^a^	1.07	1.08
**Male**	—	1.48^a^	1.46^a^	—	0.93	0.93
**Age**	—	1.06^a^	1.06^a^	—	1.05^a^	1.05^a^
**Parent education**						
<High school diploma [Reference]	—	1.00	1.00	—	1.00	1.00
High school diploma	—	0.97	0.97	—	1.02	1.07
Some college	—	0.85	0.86	—	0.84	0.90
College diploma	—	0.68^a^	0.69^a^	—	0.67^a^	0.74^a^
>College diploma	—	0.58^a^	0.59^a^	—	0.52^a^	0.59^a^
**Parent is obese**	—	2.43^a^	2.44^a^	—	2.47^a^	2.48^a^
**Neighborhood characteristics^c^**						
Neighborhood collective efficacy^d^	—	—	1.02	—	—	1.02
Neighborhood poverty^e^	—	—	1.06	—	—	1.17^a^
Neighborhood mobility^f^	—	—	0.99	—	—	0.96
**Urbanicity**						
Suburban [Reference]	—	—	1.00	—	—	1.00
Rural	—	—	1.04	—	—	1.01
Urban	—	—	0.87	—	—	0.88
**Perceived stress *z* score^c^^, ^ g**	—	—	0.98	—	—	1.00
**Constant**	1.78	0.63	0.70	0.54	0.25	0.24

a *P* < .01.

b *P* < .05.

c Calculated as *z* scores, normed such that the mean equals zero and standard deviation equals 1. Coefficients can be interpreted as the adjusted odds ratio associated with a 1 standard deviation increase in the predictor.

d A measure of social cohesion scored on a scale of 0 to 3, with a higher score indicating better neighborhood efficacy.

e Percentage of people living below the official poverty threshold, based on 1990 Census block group data.

f Measured as the percentage of occupied housing units into which people moved during the previous 5 years, based on1990 Census block group data.

g Measure of stress based on the Cohen Perceived Stress Scale (PSS) (17,18). Scored on a scale of 0 to 16, with higher scores indicating greater stress.

## Discussion

Studies investigating social determinants or structural risk factors and the incidence of type 2 diabetes, overweight/obesity, and obesity are increasingly common (3,10–13,19). Numerous studies examined social determinants or structural risk factors such as the built environment or neighborhood surroundings and their associations with such health outcomes as obesity and type 2 diabetes among racial/ethnic minority populations (20,21). However, unlike other studies, our research investigated social determinants or structural risk factors that might explain the higher incidence of type 2 diabetes, overweight/obesity, and obesity among American Indians. Type 2 diabetes, overweight, and obesity are growing health concerns for American Indian adolescents and young adults. Consistent with findings from previous studies, our study provides evidence that American Indian young adults have higher rates of elevated HbA1c levels, self-reported type 2 diabetes or high blood glucose, and overweight/obesity or obesity than have non-Hispanic whites (1,3). American Indians in our subsample also had higher rates of risk factors for poor health: they were more likely to live in neighborhoods with higher rates of poverty and housing mobility than the full sample. In addition, American Indians had higher rates of perceived stress.

Controlling for other variables, American Indian race/ethnicity was positively associated with a greater likelihood of elevated HbA1c (compared with non-Hispanic whites), and overweight/obesity and obesity partially mediated elevated HbA1c. These results are consistent with previous research (22,23). Only one of our neighborhood measures was significantly associated with HbA1c, and it was not associated in the hypothesized direction: greater neighborhood collective efficacy predicted higher HbA1c. Previous research demonstrated an association between higher collective efficacy and decreased risk of obesity and overweight among adolescents (24). However, our Census measures were based on 1990 statistics, whereas other Wave 1 data were collected in 1994–1995. Future research should continue to explore these potential links, using more precise neighborhood indicators.

American Indians were also more likely than non-Hispanic whites to report high blood glucose or diabetes. The inclusion of control and risk factors did not mediate these associations, with the exception of stress. Perceived stress was a significant mediator of the likelihood of self-reported high blood glucose or diabetes among American Indians. Research suggests that stress may influence the onset of type 2 diabetes (25).

Consistent with other findings, neighborhood characteristics such as poverty were associated with an increased risk of high BMI in non–American Indian population groups (10,26–28). Controlling for other variables, American Indians in our study were more likely to be overweight or obese than non-Hispanic whites; high BMI among American Indians was partially mediated by neighborhood poverty. Neighborhood poverty is a risk factor for poor health; future research should examine other factors associated with neighborhood poverty, such as access to grocery stores, safety, and walkability, among American Indians (19,20,21,26).

Although our study focused on the American Indian subsample, it is also interesting to compare our findings on American Indians and blacks. Without any control variables, the AOR for elevated HbA1c was 2.66 for American Indians and 4.86 for blacks, but in the prediction of self-reported high blood glucose or diabetes, the AORs were 2.39 for American Indians and 1.48 for blacks. Because the outcomes in these models reflect *being told* by a doctor, nurse, or other health care provider that one has high blood glucose or diabetes, these findings suggest that American Indians are more likely to be screened and treated for diabetes — by the Indian Health Service or others — so that they are more aware of their diabetes risk and perhaps more likely to be managing their condition. We did not measure insulin use or health insurance coverage in our study, but those issues are important for understanding differences among racial/ethnic subgroups.

This study has several limitations. First, we could not identify causal relationships between our predictors and outcomes of interest. Despite the use of longitudinal data and a robust set of control variables, our analytic strategy did not rule out the possibility of omitted variable bias. Second, our definition of the category “American Indian” combined data on participants self-reporting solely as American Indian and data on those self-identifying as American Indian in combination with one or more other groups. Future studies could investigate these American Indian subgroups separately. Third, despite the inclusion of several social determinants of health and a perceived stress indicator that might partially explain how context affects health, we did not formally test the complex pathways linking these variables to our outcomes. Future research might employ structural equation modeling or other path analyses to explore these relationships more precisely.

Despite these limitations, our study extends knowledge via several key strengths. First, the study focuses on American Indian young adults, filling a gap in the literature (1,3,5,10–13,29). Much of the research examining diabetes among American Indians is dated and does not use data from large samples such as Add Health. Second, although we do not make any claims about causal relationships, the use of longitudinal data suggests that the associations between social determinants and health outcomes persist over time; this persistence points out the need for future research in this area. Third, our analytic approach of staging control variables demonstrates the extent to which certain risk factors may play a mediating role above and beyond other control variables. We hope that future research will build on this effort to estimate the effects of an improved set of social determinants among American Indian and Alaska Native populations, especially neighborhood and housing risk factors, such as safety and overcrowding.

This study emphasizes the need to further investigate the social determinants of overweight, obesity, type 2 diabetes, and elevated HbA1c. Our research suggests that neighborhood factors and stress partially explain elevated risk for overweight, obesity, and type 2 diabetes among American Indians and that future research should include additional neighborhood factors, such as access to grocery stores selling healthy foods, proximity and safety of playgrounds or other recreational space, and adequate housing. Because neighborhood characteristics such as social capital and perceived safety are associated with lower levels of obesity in children (10,11,20,24), future research should also examine these potentially protective factors at the individual, family, and community levels.
